# A hybrid P300-SSVEP brain-computer interface speller with a frequency enhanced row and column paradigm

**DOI:** 10.3389/fnins.2023.1133933

**Published:** 2023-03-15

**Authors:** Xin Bai, Minglun Li, Shouliang Qi, Anna Ching Mei Ng, Tit Ng, Wei Qian

**Affiliations:** ^1^College of Medicine and Biological Information Engineering, Northeastern University, Shenyang, China; ^2^Key Laboratory of Intelligent Computing in Medical Image, Ministry of Education, Northeastern University, Shenyang, China; ^3^Department of Biomedical Engineering, College of Precision Instruments and Optoelectronics Engineering, Tianjin University, Tianjin, China; ^4^Shenzhen Jingmei Health Technology Co., Ltd., Shenzhen, China

**Keywords:** brain-computer interface, speller, electroencephalography, machine learning, Neural decoding

## Abstract

**Objective:**

This study proposes a new hybrid brain-computer interface (BCI) system to improve spelling accuracy and speed by stimulating P300 and steady-state visually evoked potential (SSVEP) in electroencephalography (EEG) signals.

**Methods:**

A frequency enhanced row and column (FERC) paradigm is proposed to incorporate the frequency coding into the row and column (RC) paradigm so that the P300 and SSVEP signals can be evoked simultaneously. A flicker (white-black) with a specific frequency from 6.0 to 11.5 Hz with an interval of 0.5 Hz is assigned to one row or column of a 6 × 6 layout, and the row/column flashes are carried out in a pseudorandom sequence. A wavelet and support vector machine (SVM) combination is adopted for P300 detection, an ensemble task-related component analysis (TRCA) method is used for SSVEP detection, and the two detection possibilities are fused using a weight control approach.

**Results:**

The implemented BCI speller achieved an accuracy of 94.29% and an information transfer rate (ITR) of 28.64 bit/min averaged across 10 subjects during the online tests. An accuracy of 96.86% is obtained during the offline calibration tests, higher than that of only using P300 (75.29%) or SSVEP (89.13%). The SVM in P300 outperformed the previous linear discrimination classifier and its variants (61.90–72.22%), and the ensemble TRCA in SSVEP outperformed the canonical correlation analysis method (73.33%).

**Conclusion:**

The proposed hybrid FERC stimulus paradigm can improve the performance of the speller compared with the classical single stimulus paradigm. The implemented speller can achieve comparable accuracy and ITR to its state-of-the-art counterparts with advanced detection algorithms.

## 1. Introduction

The brain-computer interface (BCI) is a communication system that does not rely on peripheral nerves and muscles to send information and commands to the outside world (Wolpaw et al., 2000). The BCI system can directly control and communicate with other entities by reading and transducing brain signals. By providing patients with conditions such as amyotrophic lateral sclerosis (ALS) and locked-in syndrome (LIS) with a way to restore their communication, the BCI system can help patients restore certain motor functions, improve their quality of life, and even enable them to enjoy life as healthy people do. Meanwhile, electroencephalography (EEG) signals are secure, non-invasive, easy to use, easy to collect, and time-resolving, making them ideal for BCI systems. At present, various BCI systems based on EEG have been developed, such as the speller systems (Farwell and Donchin, 1988; Cecotti, 2010), wheelchair control systems (Li J. et al., 2013; Li Y. et al., 2013; Li et al., 2014; Kaufmann et al., 2014), prostheses, and mechanical arm control systems (Pfurtscheller et al., 2010; Hochberg et al., 2012; Onose et al., 2012).

Electroencephalography-based BCI systems can be divided into two types: spontaneous or evoked. A representative of spontaneous BCI is Motor Imagery (MI), in which the user autonomously controls their thought activity to form certain identifiable potential information to control an external device (Jiang et al., 2020; Chen et al., 2021; Pei et al., 2021). However, the user often needs sufficient training to become proficient. The representatives of evoked BCI systems are based on the event-related potential (ERP) and steady-state visually evoked potential (SSVEP). These BCIs are characterized by the need to rely on external stimuli to evoke certain specific potential information in the human brain. However, the advantage is that the evoked signals are often stable, and the user generally only needs to know the basic operation process, so evoked BCIs have received more attention, and their technology is relatively mature.

Event-related potential is a transient characteristic potential evoked by a small probability event (Squires et al., 1975), and the most commonly detected potential is the P300 potential. This is excited as a positive voltage approximately 300 ms after the onset of the target stimulus (Picton, 1992; Luck and Kappenman, 2011). In the “Oddball” paradigm, this component is evoked when a rare stimulus (target stimulus) appears in between several relevant stimuli (non-target stimulus). The task of the subjects was to focus on the target stimulus and count its occurrences (Sur and Sinha, 2009). The P300-based speller systems have been proposed early on, and most of them are based on the “Oddball” paradigm. The earliest P300 speller system using the row and column (RC) paradigm was proposed by Farwell and Donchin (1988), whose paradigm is in the form of a 6 × 6 matrix. Rows and columns are blinked once in each stimulus trial in a random order to induce the P300 signal, and the system determines the user’s target character (intersection of rows and columns) by determining which row and which column triggered the P300 signal. Most previous studies have increased their accuracy by superimposed averaging of EEG signals, but resulting from the inefficiency, single-trail recognition of P300 has become a hot topic.

Steady-state visually evoked potential is a periodic response induced by a stimulus at a specific frequency. When the subject looks at a target flashing at a specific frequency, the subject’s EEG signal is significantly enhanced, and significant peaks can be observed at the harmonics of the frequency after the time-to-frequency conversion. Meanwhile, it was shown that the SSVEP frequency located in the center of the visual field has the most pronounced energy increase and gradually decays toward the periphery in an approximately Gaussian distribution (Sutter, 1992) so that the subject needs to gaze at the stimulus target constantly. In addition to frequency, the phase can encode the stimulus frequency (Chen et al., 2014) and the single-stage paradigm. This can present many targets simultaneously and is applied in the design of SSVEP-based spellers. However, in later studies, the Multi-stage paradigm (Multi-stage) proved more prevalent and efficient (Kick and Volosyak, 2014; Li et al., 2021). Cecotti (2010) proposed a multilevel paradigm-based SSVEP speller system, while Nakanishi et al. (2014) proposed a hybrid frequency and phase coding for a frequency and phase-based SSVEP speller system. Chen et al. (2014) proposed a stimulus paradigm with 40 target characters and compared two frequency-phase mixing patterns.

Many studies have been performed to improve the performance and create a hybrid BCI paradigm. Since both SSVEP and P300 are EEG signals and their detection regions are independent of each other, that is, they come from the time and the frequency domain, respectively, it is feasible to design hybrid triggered systems combining P300 and SSVEP without additional data acquisition equipment (Xu et al., 2016; Kundu and Ari, 2022). Panicker et al. (2011) first introduced a hybrid P300/SSVEP BCI, which uses a P300 signal for target detection and an SSVEP signal for asynchronous spelling control, and only starts P300 spelling when SSVEP reaches a certain threshold. Xu et al. (2013) developed a hybrid BCI speller that, with the same target stimulus, evokes P300 spelling in different ways to evoke both P300 and SSVEP blocking (SSVEP-B). Yin et al. (2013) have developed a hybrid BCI speller system that divides the conventional P300 speller into six groups, each flashing at a different frequency, that combines distinct features of P300 and SSVEP to reduce the number of errors in the same row or column relative to the target errors that occur. More recently, a few hybrid BCI speller systems were constructed (Kapgate et al., 2020; Xu et al., 2020; Katyal and Singla, 2021; Han et al., 2022). Han et al. (2023) even developed one high-speed system with over 200 targets, greatly expanding the instruction set.

It is noted that there is a significant competing effect when P300 and SSVEP visual stimuli are presented simultaneously; SSVEP stimuli will reduce the amplitude of the P300 signal, while P300 stimuli significantly reduce the band power of the SSVEP signal. However, as reported in the previous study, this competing effect will not result in a significant decrease in decoding accuracy because the extracted features from the reduced signals are still able to discriminate between target categories (Lee et al., 2018).

In this study, we designed and implemented a hybrid BCI speller system with high compatibility and scalability. The contributions are in four aspects. First, a frequency enhanced row column (FERC) paradigm is proposed as a new hybrid stimulus paradigm. Second, the frequency coding is incorporated into the RC paradigm so that P300 and SSVEP signals can be evoked simultaneously. Third, the new hybrid P300-SSVEP speller outperforms that only using P300 or SSVEP. Fourth, advanced detection algorithms of P300 and SSVEP and their further fusion are done. The remainder of this work is organized as follows. Section “2. Experimental methods” describes our methodology, the experimental dataset used in this work, and the corresponding data processing methods. Section “3. Results” describes the results. Section “4. Discussion” gives our conclusion and provides a discussion.

## 2. Experimental methods

### 2.1. The P300/SSVEP hybrid stimulation paradigm

**FIGURE 1:**
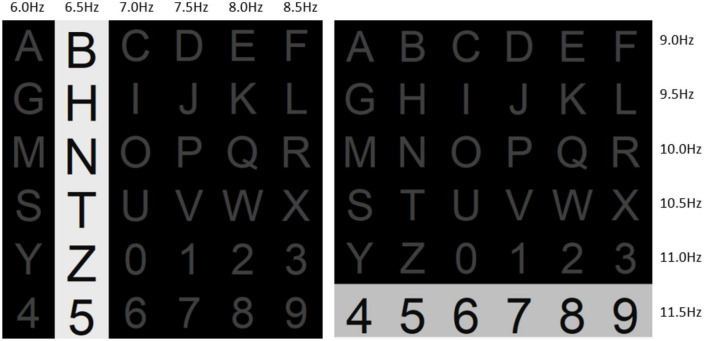
The proposed frequency enhanced row and column stimulus paradigm. A flicker (white-black) with a specific frequency is assigned to one row or column of a 6 × 6 layout, and the row/column flashes are carried out in a pseudorandom sequence. In this figure, Column 2 and Row 6 are activated, and the target output is the number “5”.

A new P300/SSVEP hybrid stimulation paradigm is proposed and named the FERC paradigm. Figure 1 shows that the stimulation interface is divided into six rows and six columns of 36 characters or targets (A to Z, 0 to 9). Each symbol is displayed equally spaced. To induce the P300 signal, the FERC paradigm uses the same principle as the Oddball paradigm, where rows and columns flash alternately in random order within a trial. Each row or column will only flash once in the same trial.

Simultaneously, to induce the SSVEP signal, the rows and columns were encoded with different frequencies of stimuli with continuous flashing. The frequency ranged from 6.0 to 11.5 Hz with an interval of 0.5 Hz, and there were 12 frequency groups. Specifically, the frequency of columns is 6.0, 6.5, 7.0, 7.5, 8.0, and 8.5 Hz; the frequency of rows is 9.0, 9.5, 10.0, 10.5, 11.0, and 11.5 Hz. For any row or column, the flashing process lasts one second. In this way, once the classifier recognizes the column and row, the target can be determined. Although the information of two stimuli is used, these stimuli are applied simultaneously in one trial. Therefore, the FERC paradigm belongs to the one-stage rather than the multi-stage paradigm.

In the stimulus interface, all characters are gray, and the background is black when there is no target flashing. The font size of characters will be enlarged by 16.67% (from 60 to 70), and their background intensity will change according to the time when the stimulus flashes appear.


(1) $$I\,n\,t\,e\,n\,s\,i\,t\,y=\frac{\text{Cos}\,\left(2\,\pi \times f\times t\right)+1}{2}$$

where *f* is the frequency of the stimulus and *t* is the duration of the visual stimulus. The reason for taking the cosine function is to ensure that the background intensity of the target character constantly changes periodically at any moment of the screen refresh and is not subject to frame loss because of the phase difference between the screen refresh and the operation.

### 2.2. Subjects

Eleven healthy volunteers (male, 19–24 years old, mean 20.7 years old) participated in our experiment, and data from 10 subjects were analyzed. All subjects provided written notification permission. They had no history of eye problems or neurological disorders, and nine subjects had no experience with the BCI system. The Medical Ethics Committee of Northeastern University approved this study. The participants provided their written informed consent to participate in this study. Before the start of the experiment, subjects were asked to minimize eye movements and to sit comfortably in a chair facing the screen.

### 2.3. Data acquisition

In this study, an actiCHamp EEG signal amplifier from Brainproducts (actiCHamp 32ch, Gilching, Germany), was used, along with the accompanying EasyCap electrode cap, which has an electrode setup in the cap corresponding to the internationally accepted standard 64 leads and a signal sampling rate of 250 Hz. In some other studies, only single-channel EEG signals have been used for BCI detection (Gao et al., 2003), but we found that multichannel data would not only yield better and more stable performance. The multichannel method is convenient for BCI applications because all users can use the same electrode cap (Bin et al., 2009).

**FIGURE 2:**
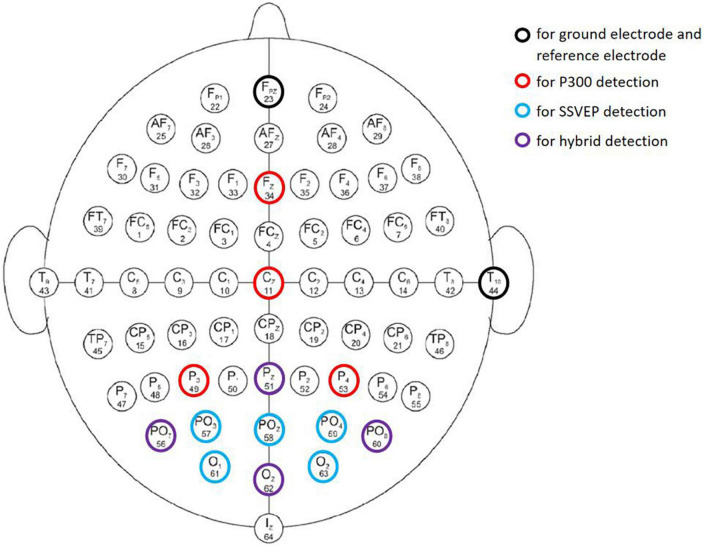
Schematic diagram of arrangements of the 15 electrodes used in the experiment.

In practice, 15 electrodes were used, including one ground electrode and one reference electrode (Figure 2). The ground electrode is located in the FPz of the international standard electrode, and the reference electrode is located in TP10 of the International standard electrode. The other electrodes were used to collect EEG signals. Fz, Cz, P3, P4, PO7, PO8, Pz, and Oz were used to collect P300 signals located in the parietal and occipital regions. Nine electrodes, PO7, PO8, Pz, Oz, O1, O2, PO3, PO4, and POz, were used to collect SSVEP signals in the occipital region. It is known from previous studies that P300 signals are mostly collected from the parietal and occipital regions (Luck and Kappenman, 2011) and SSVEP signals are mostly collected from the occipital region (Allison et al., 2014). Therefore, we selected the most helpful electrodes for the experimental results from the above regions and finally determined the above 15 electrodes.

The stimulus is displayed on a 27-inch monitor with a refresh rate of 240 Hz. All operational analysis is carried out in Matlab 2018b. After the collection, the data will be sent to Matlab software in the data processing equipment through TCP/IP protocol by the Remote Data Access (RDA) module, and the marked data will be shunted.

### 2.4. Signal preprocessing

There are four main steps in the preprocessing stage of EEG signals. Firstly, the raw EEG signal was filtered with a 60 Hz notch filter in order to remove the effects of the industrial frequency interference. Secondly, the filtered data were corrected to the baseline by “Detrending.” Thirdly, the data were cut into approximately 0–800 ms segments after stimulus onset. Finally, the data were divided into two channels, P300 and SSVEP, and entered into the next step of the experiment.

### 2.5. Feature extraction and classification

**FIGURE 3:**
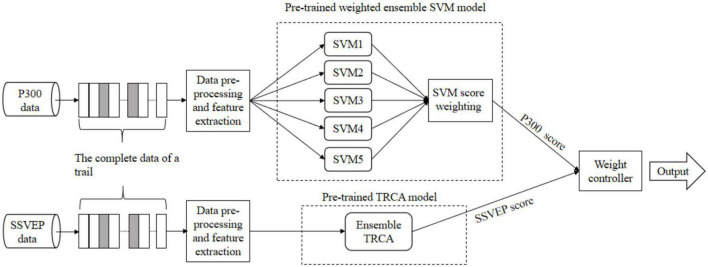
The data processing process for a single trial in the hybrid speller system. The gray blocks in the data section indicate target stimuli, and the white blocks indicate non-target stimuli. The two models and one weight controller are pre-trained by the calibration data.

The data processing procedure for a single trial in our speller system is given in Figure 3. The triaged P300 and SSVEP data are passed into their respective data processing modules and analyzed separately with different processing methods to extract the feature vectors. The processed P300 data are passed into the five support vector machine (SVM) classifiers trained in advance, and then the classification results are fed into the modified weight controller to derive the P300 score. For SSVEP data, they are fed into the pre-trained task-related component analysis (TRCA) model at the end of the pre-processing procedure to derive the SSVEP score. The two scores are passed into the total weight controller to get the final output.

#### 2.5.1. P300 feature extraction and classification

The superimposed averaging helps to improve the signal-to-noise ratio of P300 but often implies a more extended output character period, which is very detrimental to the efficiency of online systems. Therefore, a single detection of the P300 signal is chosen to improve the overall efficiency. It is well known that detecting single-trial P300 is a complex problem, so we used a follow-up operation to improve the accuracy.

The pre-processed data were used for feature extraction by wavelet transform. Since the frequency domain of the P300 signal ranges from 0.3 to 15 Hz, which is a low-frequency signal, the original signals are decomposed to a frequency scale of 0–12.5 Hz by wavelet transform. The *m* low-frequency wavelet coefficients of *n* channels are concatenated to form a feature vector of length *m* × *n* as the features of P300 and fed into the classifier for classification. Here, *m* and *n* are 13 and 8, respectively.

After the feature extraction, the SVM method is adopted to detect the occurrence of P300 because of its better generalization ability compared with other machine learning algorithms (Theodoridis and Koutroumbas, 2006). The principle of SVM is to find the hyperplane with the maximum distance that can separate sample categories from the high-dimensional space after mapping samples to the high-dimensional space. The Gaussian kernel is selected as the kernel function of SVM, which mainly considers the unique advantages in solving nonlinear problems when we are uncertain whether the P300 single detection problem is linearly separable.

Considering the single detection of the P300 signal, a weighted ensemble SVM method is further proposed to counteract the instability of the individual classifier to enhance the classification accuracy. Determined by the proposed paradigm in this study, the ratio of Target Stimulus Signal (TSS) samples containing the P300 signal to Non-target Stimulus Signal (NSS) samples in the collected EEG data is 1:5. Therefore, an undersampling method similar to the “EasyEnsemble” algorithm is adopted. Specifically, the NSS samples are randomly and evenly divided into five parts, the TSS samples are copied five times, and one part of NSS samples and one copy of TSS samples are combined into a training set to train one SVM classifier. In this way, the problem of sample imbalance is transformed into a problem of combining five classifiers (SVM1, SVM2, …, SVM5) with different training sets, which requires ensemble learning techniques.

The core idea of ensemble learning is to combine multiple individual learners to solve the same problem. A modified weighted ensemble method is used based on the theory of ensemble learning and the actual situation of the paradigm used in this study. It can dynamically adjust the weights of the classifiers according to the actual situation.

Suppose there are n trained classifiers (classifier_1_, classifier_2_, ……, classifier_n_), where (n > 0) and their cross-validation accuracies are (acc_1_, acc_2_, ……, acc_n_), let their weights be (*w*_1_, *w*_2_, ……, *w*_n_) respectively, and the sum of the weights is 1. To make the weight of the classifiers with high accuracy greater, one can make their weights proportional to the accuracy distribution.


(2) $$\left\{ \begin{array}{c} \frac{w_{1}}{a\,c\,c_{1}}=\frac{w_{2}}{a\,c\,c_{2}}=\frac{w_{3}}{a\,c\,c_{3}}=\ldots =\frac{w_{n}}{a\,c\,c_{n}}\\ \sum_{i=1}^{n}w_{i}=1 \end{array}\right.$$

Then for any m ∈[1, n], the system of equations has the following solutions.


(3) $$w_{m}=\frac{a\,c\,c_{m}}{\sum_{n}^{i=1}a\,c\,c_{i}}$$

However, suppose weights are assigned directly according to Equation 3. In that case, there is the problem that classifiers with better performance do not receive higher weights, which may make the result after integration inferior to using a single sub-classifier with better performance and render the integration useless. Therefore, this study improves on Equation 3 by introducing a theoretical accuracy to correct the weights to ensure the performance of the integration method.

Knowing the accuracy *P* of the classifier in the case of completely random target selection, we can assume that the classifier with classification accuracy *acc*_*i*_ > *P* plays a positive role. The classifier with *acc*_*i*_ < *P* is ineffective and cannot play a role, and the classifier with *acc*_*i*_ = *P* plays a negative role. Based on this conclusion, we improve the set of equations in Equation 2 and obtain the following equation.


(4) $$\left\{ \begin{array}{c} \frac{w_{1}}{a\,c\,c_{1}-P}=\frac{w_{2}}{a\,c\,c_{2}-P}=\frac{w_{3}}{a\,c\,c_{3}-P}=\ldots =\frac{w_{n}}{a\,c\,c_{n-P}}\\ \sum_{i=1}^{n}w_{i}=1 \end{array}\right.$$

For any m∈[1, n], *w_m_* can be calculated by


(5) $$w_{m}=\frac{a\,c\,c_{m}-P}{\sum_{n}^{i=1}\left(a\,c\,c_{i}-P\right)}$$

According to the modified assignment method, the weights of those with higher classification accuracy are effectively scaled up, which is more helpful in improving the minimum level of the integrated classifier and saving it from being affected by the classification performance of classifiers with low accuracy.

#### 2.5.2. SSVEP feature extraction and classification

For the SSVEP data, the filter bank technique is utilized first. The filtered data are input in the TRCA model pre-trained by the calibration data. Ten sub-bands (Sub1–Sub10) are set as Sub(k): [k × fa, 90] Hz, k = 1, 2,…, 10. To avoid distortion resulting from bandpass filtering, each sub-band filter has an extra width of 2 Hz on the low-frequency side, such as the actual sub-bands have a lower frequency limit of (k × fa − 2) Hz. Subsequently, a set of bandpass filters for these sub-bands are designed, and the Chebyshev I-type bandpass filter is used. The filter bank processing enhances the relative strength of the stimulus frequency harmonics, improving its detection accuracy and making the harmonics of the stimulus frequency much more usable for SSVEP detection. This data up-dimensioning method can improve the accuracy of recognition when the time required for calculation is not much or not limited.

The TRCA has previously been used for the BCI speller by Nakanishi et al. (2017). In our study, the calibration data of stimulus “n” used to perform SSVEP detection are defined as a four-dimension tensor x = (χ)_njkh_∈R^Nf × Nc × Ns × Nt^, and its corresponding test data or data to be detected are defined as a two-dimensional matrix X ∈ R^c × Ns^. Here n and N_f_ denote the identifier and number of target stimuli, j and N_c_ denote the identifier and number of channels, k and N_*s*_ denote the identifier and number of sampling points, and h and N_t_ denote the identifier and number of experimental trials. TRCA extracts task-relevant components by spatially filtering the training data. The spatial filter ω_*f*_ ∈ R^Nc × 1^ at the stimulus frequency *f* can be calculated by the following equation.


(6) $$a\,r\,g_{\omega_{f}}\,m\,a\,x\,\frac{\omega_{f}^{T}\,A^{T}\,A\,\omega_{f}}{\omega_{f}^{T}\,B^{T}\,B\,\omega_{f}}$$

where A ∈ R^Ns × Nc^ denotes the result of averaging over N_t_ blocks at a frequency *f* in Z.


(7) $$A=\frac{1}{N_{t}}\,\sum_{i=1}^{N_{t}}Z_{i,f}$$

B=[Z*^T^*_1,*f*_ Z*^T^*_2,*f*_ Z*^T^*_3,f_ … Z*^T^*_Nt,*f*_]^T^ ∈ R^Nt ×Ns × Nc^, and *Z*_*i*,*f*_ ∈ R^Ns × Nc^ denotes the multichannel EEG signal with stimulation frequency *f* in block *i*. After calculating the spatial filter ω_*f*_ at frequency *f*, TRCA uses the Pearson correlation coefficient between the averaged training data across trials for *n*-th visual stimulus and the test signal X ∈ R^Ns × Nc^ as the final discriminant.


(8) $$p^{\prime }=c\,o\,r\,r\,\left(X\,\omega_{f},A\,\omega_{f}\right)$$

The pre-trained TRCA model is a classifier with 12 categories.

### 2.6. Weight controller combining P300 with SSVEP

The probability of each category is obtained and represented as the SSVEP score in the form of a 12 × 1 vector. Similarly, the P300 score is also represented by a 12 × 1 vector. The weight controller combines these two scores to yield the final prediction. The weights in the controller are determined by Equations 9, 10.


(9) $$\left\{ \begin{array}{c} \frac{w_{P}}{a\,c\,c_{P}-E}=\frac{w_{S}}{a\,c\,c_{S}-E}\\ w_{P}+w_{S}=1 \end{array}\right.$$

(10) $$\left\{ \begin{array}{c} w_{P}=\frac{a\,c\,c_{P}-E}{a\,c\,c_{P}+a\,c\,c_{S}-2*E}\\ w_{S}=\frac{a\,c\,c_{S}-E}{a\,c\,c_{P}+a\,c\,c_{S}-2*E} \end{array}\right.$$

where *acc*_*P*_ and *acc*_*S*_ are the system’s recognition accuracy for the P300 and SSVEP signal, respectively; *w_P_* and *w_S_* are the weights of the kinds of signals; *E* is the accuracy of the completely randomized system.

### 2.7. Experimental setup

**FIGURE 4:**
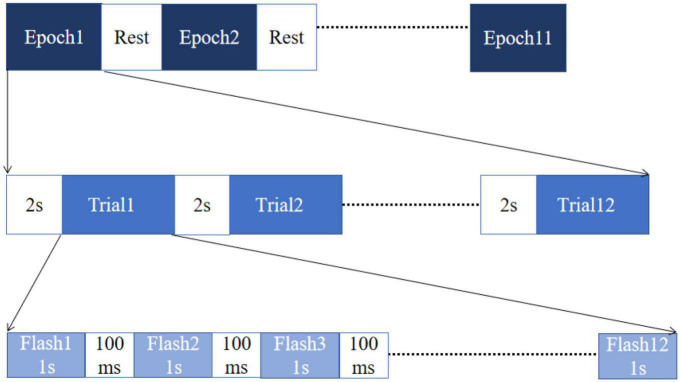
The time sequence diagram of the offline calibration.

The experimental procedure was divided into two phases for each subject: offline calibration and online testing. In the first phase, 11 epochs of data are collected, and the procedure is shown in Figure 4. During the experiment, the subjects only need to focus on their target and keep their eyes on the target location, ignoring the rest of the stimuli. Each epoch consists of 12 trials corresponding to a character input. Before each trial, two seconds are given to the subject for identifying, locating, and gazing at the target character. Each trial contains 12 flash stimuli (6 rows and 6 columns). The order and flashing frequency of the flashing stimulus were determined by the paradigm defined in section “2.1. The P300/SSVEP hybrid stimulation paradigm.” The duration of each visual stimulus flashing is 1 s, and the stimulus interval is 100 ms.

No feedback is given to subjects throughout the calibration phase. For each subject, 1,584 trials (samples) were collected. Using these samples, the two models (Pre-trained weighted ensemble SVM model and Pre-trained TRCA model) and one weight controller are trained and tested offline in a cross-validation fashion. Specifically, 10 trials of data are used for training, and 1 trial of data is used for testing with 11 cross-validations.

The second phase of the test was divided into two parts: a copy speller test and a free spelling test. In the copy speller test, subjects are asked to type “BCISPELLER,” “HELLOWORLD,” and “NEUBMIE” in sequence (27 characters in total). For each subject, 972 flashes (27 characters × 12 flashes × 3 repetitions) are collected. The EEG signal of each flash collection is called a data sample and is sequentially input to the trained models and weight controller for real-time online judgment. In the free input test, subjects are asked to spell any number of arbitrary characters and to inform the experimenter of the characters in mind at the end of the experiment, which the experimenter recorded.

**FIGURE 5:**
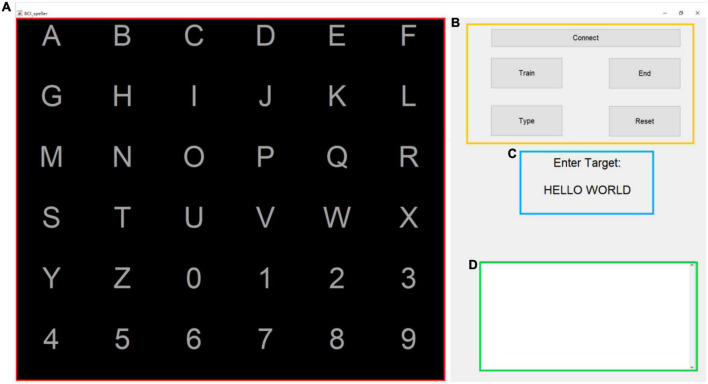
The graphical user interface used by the hybrid speller system. The area marked in red **(A)** is the visual stimulus interface; the area marked in yellow **(B)** is the control panel; the area marked in blue **(C)** is the input target prompt text box, and the area marked in green **(D)** is the input text box.

During the test phase, at the end of each trial, as real-time feedback, the characters identified from the EEG signal of that trial will be displayed on the screen. In summary, the graphical user interface used by the hybrid speller system is given in Figure 5.

In both the offline calibration and online testing phases, subjects are given a 3–7 min break between each epoch of the test to relieve visual fatigue and relax their mind to avoid the impact of nervousness or excitement on the subsequent test, with the exact duration of the break determined by the subject.

### 2.8. Evaluation and comparison

Two important metrics are usually used to evaluate the performance of a BCI speller system: accuracy (ACC) and the information transfer rate (ITR). These two metrics represent the input accuracy and speed of the system, respectively. ACC is defined as the ratio of the number of successful target selections (*X*_1_) to the total number of system inputs (*X*)


(11) $$A\,C\,C=\frac{X_{1}}{X}\times 100\%$$

The input speed and the number of characters should also be considered, so Wolpaw introduced the metric ITR, which shows the amount of information that can be transmitted in 1 min (Wolpaw, 2007)


(12) $$I\,T\,R=\left\{l\,o\,g_{2}\,N+P\,l\,o\,g_{2}\,P+\left(1-P\right)\,l\,o\,g_{2}\,\frac{1-P}{N-1}\right\}/T$$

here N is the number of categories of output commands available in the system, P is the probability of correctly selecting the target option, and T denotes the time of each trial of experiments.

The performance of the weighted ensemble SVM model for P300 and the ensemble TRCA model for SSVEP is compared with that of its three counterparts. They are FLDA (Fisher Linear Discriminant Analysis), BLDA (Bayesian Linear Discriminant Analysis), and CCA (Canonical Correlation Analysis). FLDA projects the data to lower dimensions, projects mean values of classes far apart, and the diffusion of projected data has been used for P300 detection (Panicker et al., 2011). BLDA uses regularization to prevent the overfitting of noisy data sets. With Bayesian analysis, the degree of regularization can be estimated automatically and quickly from the training data without time-consuming cross-validation (Hoffmann et al., 2008). CCA is a standard algorithm in SSVEP BCI (Lin et al., 2006; Bin et al., 2009), a multivariate statistical algorithm that attempts to reveal the correlation between two data sets. Moreover, our hybrid speller is compared with some state-of-the-art counterparts regarding the number of subjects, detection algorithms, stimulus paradigms, ACC, and ITR.

## 3. Results

### 3.1. Performance of the hybrid speller in the offline calibration tests

Table 1 presents the performance of the hybrid speller and its counterparts in the offline calibration tests, the standard deviation of ACC and ITR is also given for different classification methods. Among the 10 subjects, the hybrid speller accuracy ranges from 93.89 to 99.31%, and the mean reaches 96.86%, much higher than that only by P300 (75.29%) and only SSVEP (89.13%). Meanwhile, the hybrid speller yields a mean ITR of 30.08 bits/min, outperforming P300 only (19.36 bits/min) and SSVEP only (25.83 bits/min). The speller by SSVEP alone outperforms P300 alone.

**TABLE 1:** Performance of the hybrid speller and its counterparts in the offline calibration tests.

Subject	ACC (%)			ITR (bits/min)		
	P300	SSVEP	Hybrid	P300	SSVEP	Hybrid
S1	76.51	87.12	95.33	19.86	24.72	29.11
S2	75.00	95.83	98.33	19.23	29.41	31.01
S3	75.00	80.42	93.89	19.23	21.58	28.28
S4	74.31	87.50	98.03	18.94	24.91	30.81
S5	72.92	96.67	99.31	18.36	29.93	31.72
S6	75.00	87.50	95.14	19.23	24.91	29.00
S7	74.31	88.75	97.50	18.94	25.53	30.46
S8	79.17	89.17	96.53	21.02	25.75	29.84
S9	76.37	86.25	96.80	19.81	24.29	30.01
S10	74.31	92.08	97.78	18.94	27.28	30.64
Mean (SD)	75.29 (1.63)	89.13 (4.51)	96.86 (1.58)	19.36 (0.69)	25.83 (2.35)	30.08 (0.99)

### 3.2. Performance of the hybrid speller in the online tests

In the online tests, our hybrid speller achieves a mean accuracy of 94.29% and a mean ITR of 28.64 bits/min (Table 2). One out of 10 subjects has an accuracy of 100% at an ITR of 32.31 bits/min, and nine subjects have an accuracy higher than 90%. In addition, all subjects had an ITR greater than 25 bits/min.

**TABLE 2:** Performance of the hybrid speller in the online tests.

Subject	ACC (%)	ITR (bits/min)
S1	88.89	25.61
S2	94.90	28.86
S3	90.74	26.56
S4	92.59	27.56
S5	100.00	32.31
S6	98.15	30.89
S7	93.75	28.20
S8	90.48	26.43
S9	95.31	29.10
S10	98.15	30.89
Mean (SD)	94.29 (3.51)	28.64 (2.08)

**FIGURE 6:**
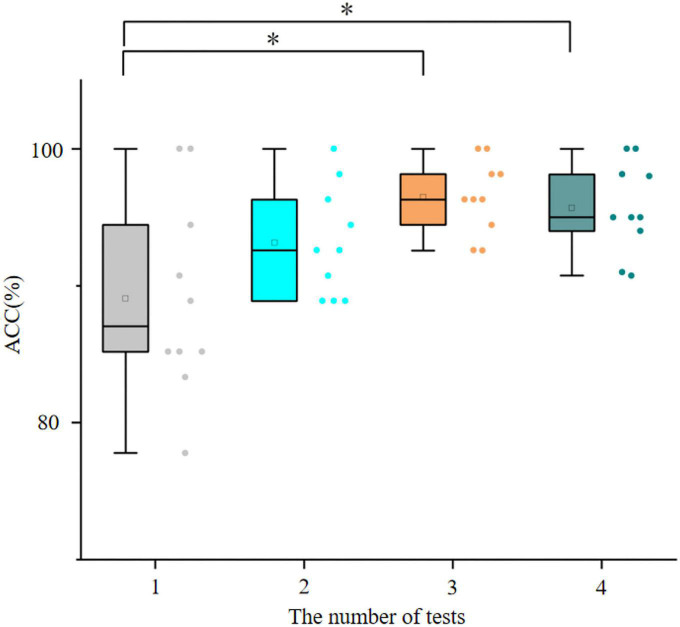
The accuracy of the hybrid speller with the number of tests in the online tests. Asterisk represents a significant difference (*p* < 0.05), 1–3 are the copy speller tests, and 4 is the free spelling test.

Figure 6 shows the accuracy of the hybrid speller with the number of tests in the online testing phase. In the copy speller tests, the accuracy increases with the number of tests, and the accuracy in the copy speller test 3 (mean value, 96.48%) is significantly higher than that in the copy speller test 1 (mean value, 89.07%). This may indicate that most subjects become increasingly proficient in using the hybrid speller system as the number of tests increases and the learning and training have performance-enhancing effects for speller users.

Interestingly, in a few cases, spelling the same character too much caused a decrease in accuracy. For example, for S5, the average accuracy of three copy speller tests is 100, 98.14, and 96.30%. The possible reason might be visual fatigue.

The free spelling test yields higher accuracy than the copy speller test 1 (89.07 versus 95.69%, *p* < 0.05). There is no significant difference between the free spelling test and the second and third copy speller tests (*p* > 0.05). This situation may be related to the subjects’ attention; specifically, too much input from the same character causes inattention, and free input may encourage the subjects to focus more actively on the target.

Many studies present the difference in the average accuracy and average ITR between the online and offline experiments (Chang et al., 2016; Katyal and Singla, 2021). In line with our results, the online performance is worse than the offline. The reason for this difference is unclear. We speculate that the performance might be affected by the subjects’ emotional state at different times, the wearing of soft electrode caps, and other issues. It is noted that the differences are within acceptable limits.

**FIGURE 7:**
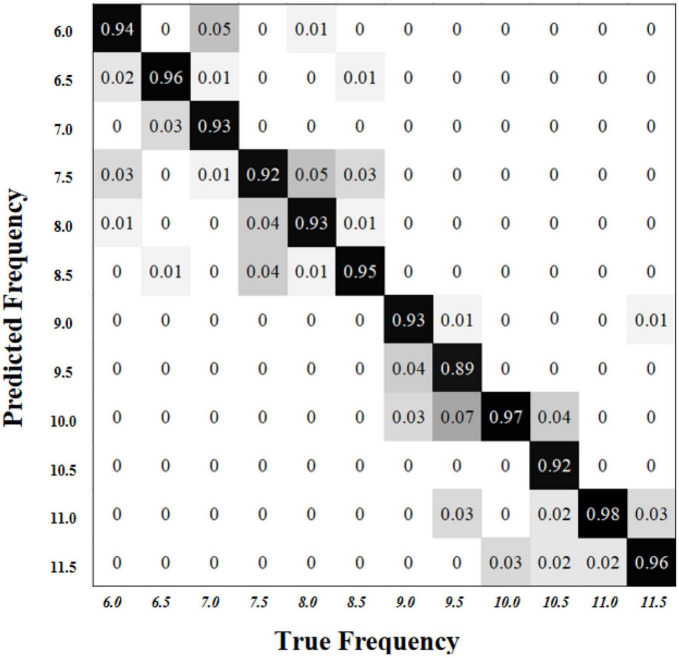
The confusion matrix in the online experiments to compare the recognition accuracy of different targets.

Figure 7 shows the corresponding confusion matrix in the online experiments to compare the recognition accuracy of different targets. The diagonal line in this matrix shows the accuracy when the predicted value is true. Because the rows and columns are calculated separately, there are no elements in the upper right and lower left parts of the confusion matrix. In most cases (10/12), the wrong identification usually occurs at the adjacent targets. Here is an example: the highest 3% error appears in the adjacent column (the third column) while the real target is located in the second column with a frequency of 6.5 Hz. There are only two special cases (the first column and the third row) and the highest error does not appear within two adjacent targets.

### 3.3. Results of the comparative experiments and spellers

Using the same dataset collected in our calibration tests, three comparative algorithms are compared with our SVM and TRCA methods (Table 3). Our SVM method achieves higher ACC compared with FLDA and BLDA in P300 detection (75.29 versus 72.22 and 61.90%) and higher ITR (19.36 versus 18.08 and 14.11). Our TRCA outperforms CCA in SSVEP detection (ACC: 89.13 versus 73.33%; ITR: 25.83 versus 18.53).

**TABLE 3:** Performance comparison of the proposed algorithm and its counterparts.

Performance	P300			SSVEP	
	FLDA	BLDA	SVM (our method)	CCA	TRCA (our method)
ACC [Mean (SD)]	72.22 (2.44)	61.90 (4.62)	75.29 (1.63)	73.33 (3.13)	89.13 (4.51)
ITR [Mean (SD)]	18.08 (1.14)	14.11 (2.89)	19.36 (0.69)	18.53 (1.87)	25.83 (2.35)

Our hybrid speller is compared with eight counterparts regarding the number of subjects, detection algorithms, stimulus paradigms, ACC, and ITR (Table 4). The number of subjects in previous studies ranged between three and twenty. The ACC is between 79.17 and 9.90%, and ITR is between 19.8 and 164.00 bits/min. Our hybrid speller performs comparably to the-state-of-art hybrid spellers (P300 and SSVEP). It is worth mentioning that we use a single-trial P300 to increase ITR and our counterparts use the method of superimposed averaging. Our hybrid speller uses a one-stage paradigm similar to the other four studies (Panicker et al., 2011; Yin et al., 2013; Xu et al., 2014; Jalilpour et al., 2020), and the other four spellers use the multi-stage paradigm (Yin et al., 2015; Chang et al., 2016; Xu et al., 2020; Katyal and Singla, 2021). The one-stage paradigm has the outstanding feature of high speed, while the multi-stage paradigm can have a higher capacity of characters (Li et al., 2021).

**TABLE 4:** Comparison of our hybrid speller to the previous ones.

Study	Key aspects	Performance
Our method	Subjects: 10; P300: SVM; SSVEP: ensemble TRCA Our paradigm causes both P300 and SSVEP signals to be hybrid by weights.	ACC = 94.29% ITR = 28.64
Panicker et al., 2011	Subjects: 3; P300: FLDA; SSVEP: CCA It uses a P300 signal for target detection and an SSVEP signal for asynchronous spelling control and only starts P300 spelling when SSVEP reaches a certain threshold.	ACC = 97.00% ITR = 20.13
Yin et al., 2013	Subjects: 12; P300: SWLDA; SSVEP: CCA SSVEP is stimulated by flashing between white and black at different frequencies, and P300 is stimulated by highlighting rows and columns using orange crosses.	ACC = 93.85% ITR = 56.44
Xu et al., 2014	Subjects: 11; P300: SWLDA; SSVEP: CCA P300 and SSVEP blocking were simultaneously evoked in different ways under the same target stimulus.	ACC = 87.80% ITR = 54.00
Jalilpour et al., 2020	Subjects: 6; P300: RLDA; SSVEP: SVM The P300 signal determines the target symbol group, and the character position is determined by the spatial characteristics of the SSVEP signal response.	ACC = 93.06% ITR = 23.41
Yin et al., 2015	Subjects: 13; P300: SWLDA; SSVEP: CCA In the multi-step SSVEP paradigm, the frequencies of SSVEP stimuli switch once midway through each selection, such as the first half of the selection is for step one and the second half for step two.	ACC = 95.18% ITR = 50.41
Chang et al., 2016	Subjects: 10; P300: SWLDA; SSVEP: CCA In the multi-stage paradigm, the flickering stimulus and periodic change of the character evoke dual-frequency SSVEP, while the oddball stimulus of the target character evokes P300.	ACC = 93.00% ITR = 31.80
Xu et al., 2020	Subjects: 10; ensemble TRCA In the multi-stage paradigm, different sub-spellers use different frequencies and initial phases, and different characters blink at different times.	ACC = 79.17% ITR = 164.00
Katyal and Singla, 2021	Subjects: 20; P300: BLDA; SSVEP: CCA In the multi-stage paradigm, P300 estimates the tier of alpha-numeric symbol sets intended by the subject. The secondary selection phase used a traditional SSVEP paradigm to elicit SSVEP markers.	ACC = 96.42% ITR = 131.00

The FERC paradigm is proposed and implemented in our speller. This paradigm differs from the four previous one-stage paradigms (Panicker et al., 2011; Yin et al., 2013; Xu et al., 2014; Jalilpour et al., 2020). To the best of our knowledge, this paradigm has not been used for the speller though a similar paradigm has been used for the BCI for other purposes (Allison et al., 2014; Wang et al., 2015).

Our work is different from previous studies mainly in two aspects: (1) the stimulation paradigm; (2) the method of feature extraction and classification of EEG signals. First, our paradigm triggers both P300 and SSVEP signals simultaneously by the flickers (white-black) of row/column with different frequencies. It is different from the previous four one-stage paradigms (Panicker et al., 2011; Yin et al., 2013; Xu et al., 2014; Jalilpour et al., 2020). Despite belonging to the same category of paradigms, they have significantly distinct features. In the paradigm of Panicker et al. (2011), P300 and SSVEP signals have different roles and P300 spelling started only when SSVEP reached a certain threshold. Yin et al. (2013) directly combined SSVEP and P300 signals triggered by different stimuli (i.e., the flashing of row/column and orange crosses, respectively). Xu et al. (2014) used the paradigm suitable for capturing signals of P300 and SSVEP blocking, while Jalilpour et al. (2020) specified the paradigm using the spatial characteristics of the SSVEP signal response. Second, for the method of feature extraction and classification of EEG signals, our work combined SVM for P300 and TRCA for SSVEP with the more advanced fusion algorithm. In previous studies, different linear discriminant analysis (LDA) algorithms [e.g, FLDA, SWLDA (step-wise LDA), RLAD (regularized LDA), BLAD] have been commonly utilized for P300 signal (Panicker et al., 2011; Yin et al., 2013, 2015; Xu et al., 2014; Chang et al., 2016; Jalilpour et al., 2020; Katyal and Singla, 2021). For SSVEP signals, the CCA method is usually adopted (Panicker et al., 2011; Yin et al., 2013, 2015; Xu et al., 2014; Chang et al., 2016; Katyal and Singla, 2021) while SVM is also used (Jalilpour et al., 2020).

## 4. Discussion

This article proposes a FERC as a new hybrid stimulus paradigm, and a hybrid speller is implemented. The frequency coding is incorporated into the RC paradigm so that P300 and SSVEP signals can be evoked simultaneously. The new hybrid P300-SSVEP speller outperforms that using P300 or SSVEP alone. Advanced detection algorithms of P300 and SSVEP (such as the weighted ensemble SVM model and the ensemble TRCA model) and their further fusion by the weight controller are crucial. In the end, the implemented hybrid speller presents a comparable performance in terms of both ACC and ITR to the-state-of-art hybrid spellers (P300 and SSVEP).

Although P300 spellers have been extensively studied and can achieve good accuracy with multiple trials per symbol, using single-trial spellings is still a challenging problem. In our study, the hybrid system incorporating SSVEP achieved an online spelling performance of 94.29% accuracy, ITR of 28.64 bits/min using a single trial, and 96.8% accuracy after two training sessions. These results show that our BCI speller is expected to enable fast spelling in stimulus-driven BCI applications.

We used data acquisition in parallel with stimulus generation and labeling for the system’s design to speed up the feedback. For the choice of paradigm, we designed the FERC to incorporate frequency coding into the RC paradigm to achieve the effect of evoking P300 and SSVEP signals simultaneously. For the selection of frequency, we chose a relatively low 6.0–11.5 Hz with a spacing of 0.5 Hz to get the best possible classification results while also considering the problem of visual fatigue of the subjects. For the page layout, we used the classic 6 × 6 layout and finally simplified all the operations into a few buttons, making it easy for people without professional training to operate.

Simultaneously, the system is not dependent on existing external platforms, so it is highly scalable and compatible, supporting many different application scenarios, and can be used within the system, in-text editing software, and social software. The application of a virtual keyboard in the feedback control module allows the system to support the input of text in various languages using the input methods already installed on the computer, meeting the user’s needs while ensuring that it is as consistent as possible with the input habits of healthy people in their lives. In subsequent performance tests, the system performed well.

The most likely explanation for the performance improvement is three aspects. First, the clever design of the hybrid stimulus mechanism can detect both signals without causing performance degradation. Second, the modified weighted integrated SVM method can classify P300 signals more efficiently compared with the traditional SVM algorithm for processing EEG signal effects. Third, the addition of the SSVEP component provides additional information that helps predict targets versus non-targets. These analyses conclude that our hybrid BCI method yields better and more stable performance than the P300-only and SSVEP-only methods.

One of the 11 subjects was discarded because of the low accuracy (33.33%). By reviewing the literature, we identified a phenomenon called “BCI Illiteracy,” which refers to the presence of a proportion of people who cannot trigger the two signal-evoked signals P300 or SSVEP, as well as the spontaneous signal MI (Lee et al., 2019). However, there is still no standardized definition of BCI blindness, so we believe this is a possible explanation.

We collected feedback from subjects after the experiment on their feelings about using the system and their experience with the experimental process and stimulation paradigm. The following two main feedback comments are obtained. First, all subjects reported significant visual fatigue during the overall length of data collection in the first phase of the experiment, and some subjects would feel annoyed or lose focus uncontrollably during the process. However, from an experimental design perspective, if the number of training sessions is reduced, it may lead to poor generalization of the model. During model calibration, we found that the accuracy of model cross-validation improved with the increase in data. Second, several volunteers reported that their attention was affected to some extent by the flickering of adjacent characters while gazing at the target characters, both during calibration and testing. They indicated that they would unconsciously shift their eyes to gaze at or look at these targets with their afterglow when the adjacent characters were illuminated, and the low accuracy of some volunteers may have been related to this factor.

Combined with the above feedback, the study of reducing the collected training data is an important future research direction in which the generated spurious data will supplement the missing training data. In addition, to further improve the performance of our BCI speller, in the future, we will conduct more research with more advanced signal processing algorithms (Pei et al., 2022), reduce the current electrode set, and select the optimal stimulus onset asynchrony for the flash frequency.

## 5. Conclusion

A hybrid BCI speller system based on a single-trial P300 and SSVEP has been designed and implemented. The frequency coding is incorporated into the RC paradigm so that the P300 and SSVEP signals can be evoked simultaneously. Advanced detection algorithms of P300 (the weighted ensemble SVM) and SSVEP (the ensemble TRCA) and their further fusion lead to good performance (average 94.8%, maximum accuracy of 100%, and ITR of 28.64 bits/min). The new hybrid P300-SSVEP speller outperforms the P300 or SSVEP alone and shows comparable performance to its state-of-the-art counterparts. These results demonstrate that our speller system has specific application prospects and practical value.

## Data availability statement

The original contributions presented in this study are included in the article/supplementary material, further inquiries can be directed to the corresponding author.

## Ethics statement

The studies involving human participants were reviewed and approved by the Medical Ethics Committee of Northeastern University. The patients/participants provided their written informed consent to participate in this study.

## Author contributions

XB performed the experiments and analyzed the data along with ML and SQ. SQ, AN, TN, and WQ conceived the study, presented the results, and wrote the manuscript along with XB. XB collected and analyzed the data. SQ and WQ supervised the algorithm development and analyzed the data. All authors read and approved the final manuscript.
